# A New Texture Spectrum Based on Parallel Encoded Texture Unit and Its Application on Image Classification: A Potential Prospect for Vision Sensing

**DOI:** 10.3390/s23208368

**Published:** 2023-10-10

**Authors:** José Trinidad Guillen Bonilla, Nancy Elizabeth Franco Rodríguez, Héctor Guillen Bonilla, Alex Guillen Bonilla, Verónica María Rodríguez Betancourtt, Maricela Jiménez Rodríguez, María Eugenia Sánchez Morales, Oscar Blanco Alonso

**Affiliations:** 1 Departamento de Electro-Fotónica, Centro Universitario de Ciencias Exactas e Ingenierías, Universidad de Guadalajara, Blvd-M. García Barragán 1421, Guadalajara 44430, Jalisco, Mexico; 2 Departamento de Farmacología, Centro Universitario de Ciencias Exactas e Ingenierías, Universidad de Guadalajara, Blvd-M. García Barragán 1421, Guadalajara 44430, Jalisco, Mexico; nancy.frodriguez@academicos.udg.mx; 3 Departamento de Ingeniería de Proyectos, Centro Universitario de Ciencias Exactas e Ingenierías, Universidad de Guadalajara, Blvd-M. García Barragán 1421, Guadalajara 44430, Jalisco, Mexico; hector.guillen1775@academicos.udg.mx (H.G.B.); veronica.rbetancourtt@academicos.udg.mx (V.M.R.B.); 4 Departamento de Ciencias Computacionales e Ingenierías, CUVALLES, Universidad de Guadalajara, Carretera Guadalajara-Ameca Km. 45.5, Ameca 46600, Jalisco, Mexico; alex.guillen@academicos.udg.mx; 5 Departamento de Ciencias Básicas, Centro Universitario de la Ciénega (CUCienéga), Universidad de Guadalajara, Av. Universidad No. 1115, LindaVista, Ocotlán 47810, Jalisco, Mexico; maricela.jrodriguez@academicos.udg.mx; 6 Departamento de Ciencias Tecnológicas, Centro Universitario de la Ciénega (CUCienéga), Universidad de Guadalajara, Av. Universidad No. 1115, LindaVista, Ocotlán 47810, Jalisco, Mexico; eugenia.sanchez@academicos.udg.mx; 7 Departamento de Física, Centro Universitario de Ciencias Exactas e Ingenierías, Universidad de Guadalajara, Blvd-M. García Barragán 1421, Guadalajara 44430, Jalisco, Mexico; oscar.blanco@academicos.udg.mx

**Keywords:** texture spectrum based on parallel encoded texture unit (TS_PETU), multi-class classifier, image classification, high efficiency

## Abstract

In industrial applications based on texture classification, efficient and fast classifiers are extremely useful for quality control of industrial processes. The classifier of texture images has to satisfy two requirements: It must be efficient and fast. In this work, a texture unit is coded in parallel, and using observation windows larger than 3×3, a new texture spectrum called Texture Spectrum based on the Parallel Encoded Texture Unit (TS_PETU) is proposed, calculated, and used as a characteristic vector in a multi-class classifier, and then two image databases are classified. The first database contains images from the company Interceramic^®®^ and the images were acquired under controlled conditions, and the second database contains tree stems and the images were acquired in natural environments. Based on our experimental results, the TS_PETU satisfied both requirements (efficiency and speed), was developed for binary images, and had high efficiency, and its compute time could be reduced by applying parallel coding concepts. The classification efficiency increased by using larger observational windows, and this one was selected based on the window size. Since the TS_PETU had high efficiency for Interceramic^®®^ tile classification, we consider that the proposed technique has significant industrial applications.

## 1. Introduction

Currently, image recognition based on the textural characteristics of images has a large number of applications in biomedicine [1,2], object detection [3,4], medical diagnostics [5,6,7], and remote sensing [8,9], among others. Because of this, around the world there is a large number of research groups that carry out research on texture analysis, extraction, and/or classification. The goal of these groups is to develop new applications whose objective is to improve quality of life.

In texture analysis, there are three main problems: texture classification, where the goal is to determine to which class a test texture belongs; texture segmentation, where the goal is to partition an image into sections based on the textures that make it up; and texture synthesis, where the goal is to generate a mathematical model in order to build the desired texture. In particular, in texture classification, the extraction of textural features is very important, according to the author of reference [10]. There are four methods to solve the problem: geometry, mathematical models, signal processing, and statistics.

The statistical method for extracting textural characteristics basically consists of selecting an observation window, the size of which is frequently W=3×3 pixels [11]. By scrolling the window pixel by pixel over the entire image, patterns are detected and encoded to calculate the texture unit [12], which we will denote as k. The unit value k depends on the encoding method, as indicated in reference [11], in which the authors describe, apply, and compare 35 different texture extraction techniques. However, in all methods, the unit k is considered a discrete variable and is used as an index in a discrete histogram h(k), whose length is from 0 to K−1. The histogram h(k) has $R^{K}$ dimensions, where the value of K is the maximum texture unit value. Now, since the objective is to apply to the histogram h(k) in image classification, it is interpreted as a texture spectrum and then is used as a characteristic vector in supervised (multi-class and one-class) and unsupervised (clustering) classifiers [10,13,14,15,16,17]. Such classification systems operate in real time and efficiently [18,19,20]; when the spectrum h(k) has low dimensional space, the histogram contains a sufficient amount of texture information of the image under study, the classifier is optimized, and the electronic device is task-specific. 

In this work, by conducting a local analysis on a binary image s(m,n) through an observation window W=I×J, an image s(m,n) is represented by a probability density function $p_{I\times J}\left(k\right)$, where m,n are the coordinates of the pixels of the digital image and k is the unit of texture. The equalized histogram $p_{I\times J}\left(k\right)$ is called Texture Spectrum Based on the Parallel Encoded Texture Unit (TS_PETU) because the unit k is coded using parallel coding concepts. Due to parallel coding, the TS_PETU histogram can be computed using larger windows at W=3×3, and as a consequence, they contain a greater amount of texture information and their dimensional space can be selected since the probability function $p_{I\times J}\left(k\right)$ has $R^{I\left(2^{J}-1\right)+1}$ dimensions. Based on the behavior of $R^{I\left(2^{J}-1\right)+1}$, two regions are defined: low-dimensional space and high-dimensional space. In the first region, the TS_PETU histogram has from $R^{22}$ up to $R^{10,231}$ dimensions and the window size is within the range of W=3×3 to W=10×10. On the other hand, in the second region, the TS_PETU histogram satisfies the condition $R^{K}>R^{10,231}$ dimension, and then the window size must satisfy I×J>10×10. 

By interpreting the TS_PETU histogram as a texture spectrum, it can be used as a feature vector in a multi-class classifier and then two image databases can be classified. The first database was acquired under controlled conditions by the Interceramic^®®^ company, and the images in the second database were acquired in natural environments. With the goal of verifying the classification efficiency of our proposal, three experiments were developed. In the first experiment, using window sizes within the range of I×J=3×3 to I×J=20×20, texture information was measured for Interceramic^®®^ tile images. Our experimental results confirm that the amount of texture information contained in the TS_PETU histogram increased due to the observation window size and its behavior was exponential, and the theoretical results are in agreement with the experimental results. In the second experiment, the TS_PETU histogram was calculated using observation windows from I×J=3×3 to I×J=20×20. It was used as a feature vector in a multi-class classifier and then the Interceramic^®®^ images were classified. Our experimental results confirm the high efficiency of the TS_PETU transform since the classification accuracy was 100%. This high efficiency is attributed to the precision with which the TS_PETU transform can work and because the images were acquired under controlled conditions. In the third experiment, using windows within the interval of I×J=3×3 to I×J=10×10, the TS_PETU histogram was calculated. Next, this was used as a characteristic vector in the classifier for multiple classes, and subsequently, the images acquired in natural environments were classified. In our results, the classification efficiency was within the range of ${Ef}_{I\times J}=84.84\%\;\left(I\times J=3\times 3\right)$ to 100% $100\%\;\left(I\times J=5\times 5\;or\;greater\right)$. The classification errors are attributed to the fact that the images were acquired under uncontrolled conditions. The increase in efficiency can be attributed to the increase in texture information due to the size of the observation window.

Based on the analysis performed on the binary image and our experimental results, the following relevant points were inferred for the TS_PETU transform: (1) The transformation has potential industrial applications, (2) its dimensional space and texture information can be selected based on the observation window size, (3) its classification efficiency improves when the observation window is larger, (4) the transformation has potential real-time application due to the parallel encoding of the texture unit, and (5) vision systems can be implemented for quality control.

## 2. Texture Spectrum Based on Parallel Encoded Texture Unit

### 2.1. Proposal Methodology

**Figure 1:**
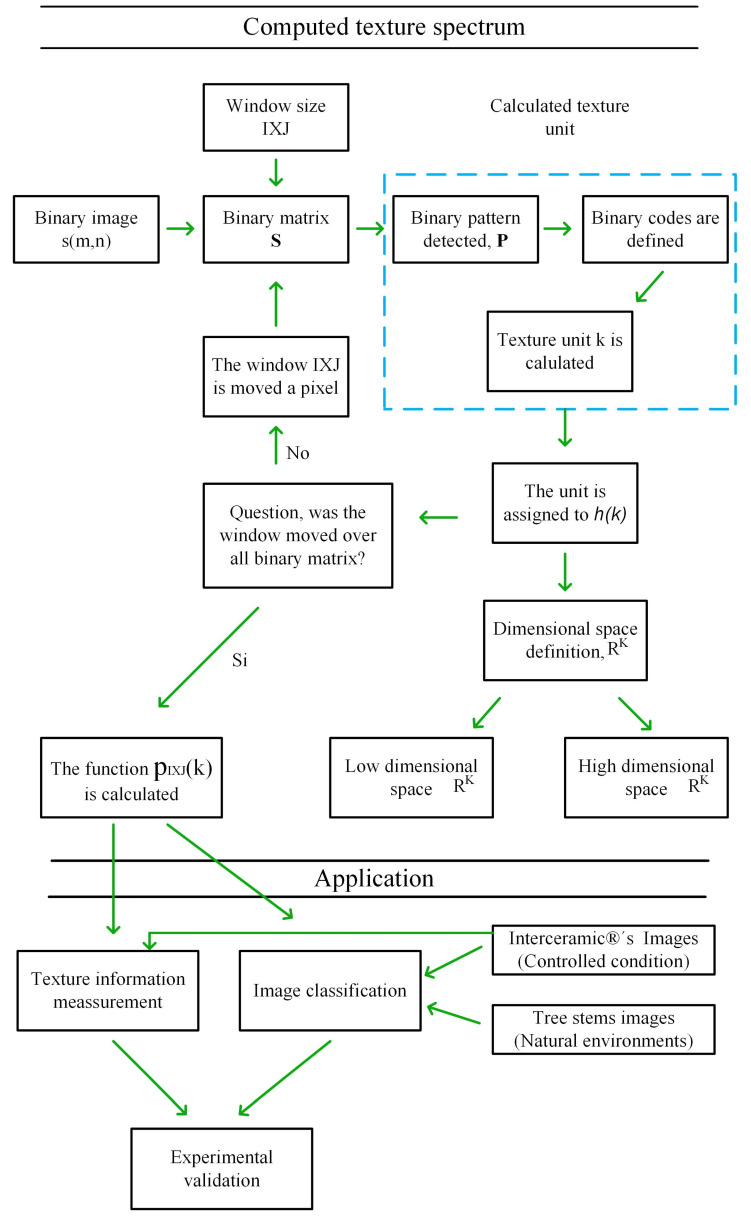
Proposed methodology for calculating the TS_PETU histogram and its application in measuring texture information and image classification: the texture unit is in blue.

Figure 1 shows the proposed procedure schematically. The methodology consists of two phases: calculation of the texture spectrum and its application. In the first phase, called texture spectrum calculation, the digital image s(m,n) is interpreted as a binary matrix $S=\left\{s_{mn}\right\}$, where the white pixels are ones and the black pixels are zeros. Once the observation window size I×J is selected, we describe the procedure to calculate the texture unit k, the encoding of which is done in parallel. Subsequently, the unit k is used as an index in the histogram $h_{I\times J}\left(k\right)$ and is also used to calculate the dimensional space $R^{K}$. Finally, an algorithm is proposed to calculate the histogram $h_{I\times J}\left(k\right)$, and with it, a probability density function is defined, $p_{I\times J}\left(k\right)$. This function is named Texture Spectrum based on the Parallel Encoded Texture Unit (TS_PETU). In the second phase, called application, the TS_PETU histogram is used to measure texture information and applied in image recognition. Both the texture measurement and classification efficiency are experimentally validated using two image databases. The first is of industrial origin and the acquisition conditions are controlled, whereas in the second database, the images were acquired in natural environments.

### 2.2. Parallel Encoded Texture Unit

With the goal of obtaining a reduced dimensional space in the texture spectrum, in this section, the texture unit is coded in parallel. The procedure is described below.

**Figure 2:**
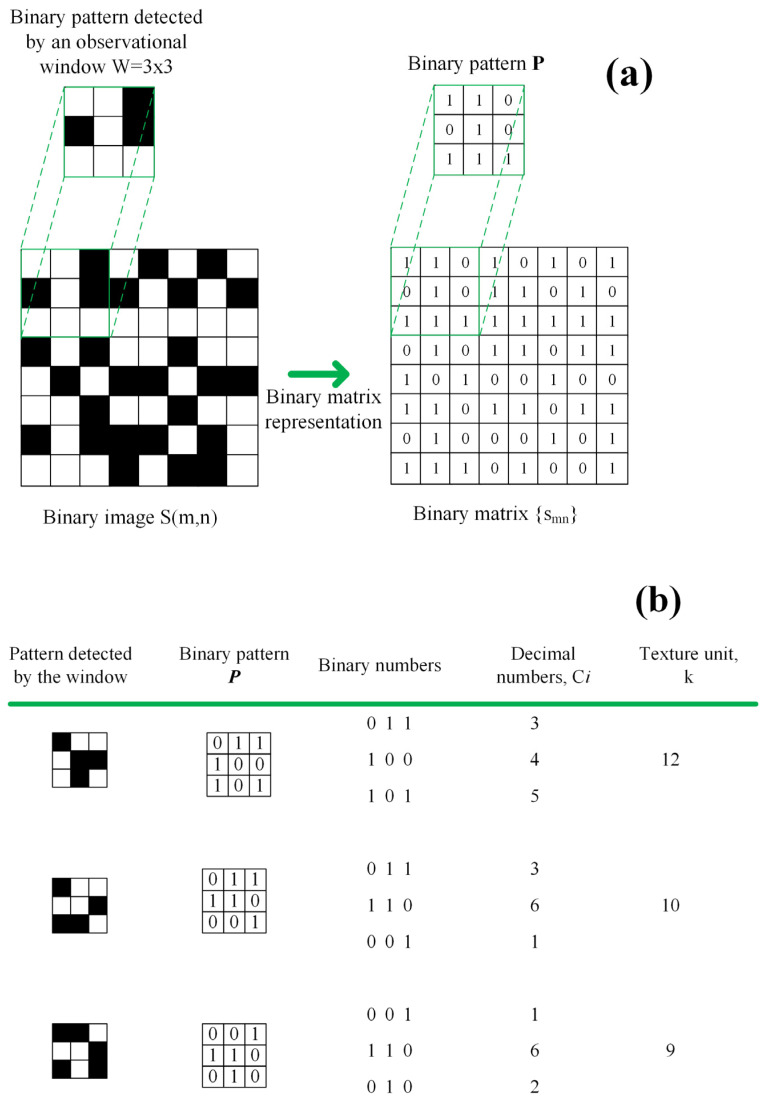
(**a**) Binary image $s\left(m,n\right)$ represented by the binary matrix $\left\{s_{mn}\right\}$: M = 8, N = 8, and the binary pattern was detected by the observational window W=3×3; (**b**) three texture unit examples calculated using the parallel codification.

Let $s\left(m,n\right)$ $\left(m=1,2,\ldots ,M;n=1,2,\ldots ,N\right)$ be a binary image with M×N pixels, which is interpreted as a binary matrix $\left\{s_{mn}\right\}$ with M rows and N columns, and let W an observation window be of the size W=I×J. Then, for each position on the matrix $\left\{s_{mn}\right\}$, the window W detects a binary pattern denoted by $P=\left[\begin{array}{cccc} a_{11} & a_{12} & \cdots & a_{1J}\\ a_{21} & a_{22} & \cdots & a_{2J}\\ \vdots & \vdots & \ddots & \vdots \\ a_{I1} & a_{I2} & \cdots & a_{IJ} \end{array}\right]$, whose number of rows is I and number of columns is J, and the number of patterns P in the matrix $\left\{s_{mn}\right\}$ is denoted by $P_{p}=(M-I+1)(N-J+1)$. This can be seen in Figure 2a, where a white pixel is 1, a black pixel is 0, and the window size is I×J=3×3.

Let P be a binary pattern detected through window W. If each row is considered a binary number, then each binary number can be independently codified as a decimal number through a BCD conversion such that the ith decimal number is calculated by means of

(1) $$c_{i}=\sum_{j=0}^{J-1}b_{j}2^{j}=b_{0}2^{0}+b_{1}2^{1}+b_{2}2^{2}+\cdots +b_{J-1}2^{J-1}$$

where $c_{i}\;(i=1,2,\ldots ,I)$ is the ith decimal number, $b_{j}$ (j=0,1,…,J−1) is the jth bit number, 2 is the base, and j indicates the jth element. Thus, the texture unit is defined by summing all decimal numbers, which are calculated from the pattern

(2) $$k=\sum_{i=1}^{I}c_{i}=c_{1}+c_{2}+\cdots +c_{I}$$

where k is our texture unit definition calculated through a parallel codification. Figure 2b shows three texture units calculated using the parallel codification I×J=3×3. Based on Figure 2, the texture unit k is calculated by carrying out the following simple procedure: (a) An observational window is defined and its size is W=I×J; (b) the window W detects a binary pattern for each position on the binary image $s\left(m,n\right)$; (c) the binary pattern is interpreted as a binary state P, whose number of rows is I and number of columns is J; (d) a decimal number is calculated from each row and there are I numbers; and (e) the texture unit k is estimated by calculating the sum of all decimal numbers.

To calculate the minimum value for the texture unit k, all elements are zeros for the pattern $P=\left[\begin{array}{ccccc} 0 & 0 & 0 & \cdots & 0\\ 0 & 0 & 0 & \cdots & 0\\ 0 & 0 & 0 & \cdots & 0\\ \vdots & \vdots & \vdots & \ddots & \vdots \\ 0 & 0 & 0 & \cdots & 0 \end{array}\right]$. Then, by applying the procedure described and considering Equation (1), decimal numbers are

(3) $$\begin{array}{c} c_{1}=0=\sum_{j=0}^{J-1}b_{j}2^{j}=0{\times 2}^{0}+0\times 2^{1}+0\times 2^{2}+\cdots +0\times 2^{J-1}\\ c_{2}=0=\sum_{j=0}^{J-1}b_{j}2^{j}=0{\times 2}^{0}+0\times 2^{1}+0\times 2^{2}+\cdots +0\times 2^{J-1}\\ \begin{array}{c} c_{3}=0=\sum_{j=0}^{J-1}b_{j}2^{j}=0{\times 2}^{0}+0\times 2^{1}+0\times 2^{2}+\cdots +0\times 2^{J-1}\\ \vdots \\ c_{I}=0=\sum_{j=0}^{J-1}b_{j}2^{j}=0{\times 2}^{0}+0\times 2^{1}+0\times 2^{2}+\cdots +0\times 2^{J-1} \end{array} \end{array}.$$

Based on Equations (2) and (3), the minimum value for the texture unit is

(4) $$k=0=c_{1}+c_{2}+c_{3}+\cdots +c_{I}=0+0+0+\cdots +0$$

On the other hand, to estimate the maximum value, all element are ones for the pattern $P=\left[\begin{array}{ccccc} 1 & 1 & 1 & \cdots & 1\\ 1 & 1 & 1 & \cdots & 1\\ 1 & 1 & 1 & \cdots & 1\\ \vdots & \vdots & \vdots & \ddots & \vdots \\ 1 & 1 & 1 & \cdots & 1 \end{array}\right]$. By carrying out the previous procedure and using Equation (1), the decimal numbers are

(5) $$\begin{array}{c} c_{1}=1{\times 2}^{0}+1\times 2^{1}+1\times 2^{2}+\cdots +1\times 2^{J-1}\\ c_{2}=1{\times 2}^{0}+1\times 2^{1}+1\times 2^{2}+\cdots +1\times 2^{J-1}\\ \begin{array}{c} c_{3}=1{\times 2}^{0}+1\times 2^{1}+1\times 2^{2}+\cdots +1\times 2^{J-1}\\ \vdots \\ c_{I}=1{\times 2}^{0}+1\times 2^{1}+1\times 2^{2}+\cdots +1\times 2^{J-1} \end{array} \end{array}.$$

From Equation (5), the following is obtained:

(6) $$\begin{array}{c} c_{1}=2^{J}\\ c_{2}=2^{J}\\ \begin{array}{c} c_{3}=2^{J}\\ \vdots \\ c_{I}=2^{J} \end{array} \end{array},$$

The texture unit is

(7) $$k=c_{1}+c_{2}+c_{3}+\cdots +c_{I}=2^{J}+2^{J}+2^{J}+\cdots +2^{J},$$

and then the maximum value of the texture unit is defined by

(8) $$K=k=I\left(2^{J}-1\right)+1$$

Based on Equations (4) and (8), the texture unit k can take a discrete value into the interval of 0 to $K-1=I\left(2^{J}-1\right)$, where K is the maximum value. This interval considers all possible states for the pattern P.

### 2.3. Dimensional Space $R^{K}$

When an image $s\left(m,n\right)$ is transformed in a texture spectrum $h\left(k\right)$ where k is the texture unit, m,n are the coordinates for the digital image, and the unit k is used as an index, the spectrum has a length of 0 to K−1 and the discrete histogram $h\left(k\right)$ has a dimensional space denoted by $R^{K}$. In the $R^{K}$ space, all possible binary states for the pattern P are considered. There are $R^{K}$ dimensions, and the maximum dimension is the maximum value of the texture unit definition. Clearly, the dimensional space is a function of the observation window size W, whose size is I×J, and therefore Equation (8) can be written as

(9) $$K(I,J)=I\left(2^{J}-1\right)+1$$

**Figure 3:**
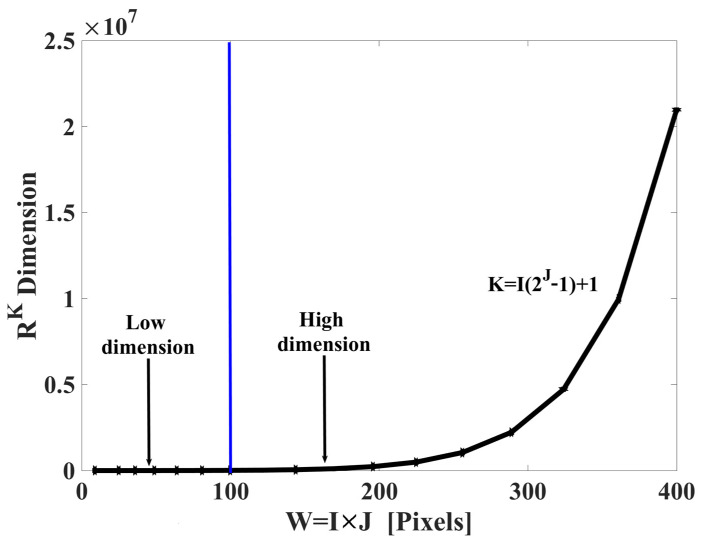
Behavior of $R^{K}\;vs.\;W=I\times J$, where I=J.

Moving forward in this work, $K\left(I,J\right)$ will be used or only K. The behavior is linear for the term I but the behavior is exponential for the term J. Therefore, the behavior of I reduces exponentially in space from $R^{2^{i\times J}}$ [12,14] to $R^{I\left(2^{J}-1\right)+1}$. In Figure 3, the behavior of $R^{I\left(2^{J}-1\right)+1}$ vs. W=I×J is observable.

By analyzing Figure 3, we can define two regions: low-dimensional space and high-dimensional space. The threshold between both regions is indicated by the blue line. In the low-dimensional space region, the observation window must be within the interval

(10) 3×3≤I×J≤10×10

and as a consequence,

(11) $$R^{22}\leq R^{K}\leq R^{10,231}$$

On the other hand, in the region of high-dimensional space, the observation window satisfies the condition

(12) I×J>10×10

and then

(13) $$R^{K}>R^{10,232}$$

is satisfied.

Computationally speaking, the texture spectrum h(k) can operate in the region of low- or high-dimensional space. This offers versatility in its application.

### 2.4. Algorithm

In texture classification, a digital image $s\left(m,n\right)$ is transformed into a discrete histogram $h\left(k\right)$, where the texture unit *k* is used as the index and the length of the discrete histogram must be within the interval of 0 to K−1. The histogram $h\left(k\right)$ is interpreted as a texture spectrum and shows the frequency of occurrence of the units calculated from the image $s\left(m,n\right)$. 

To avoid mathematical complexity, an algorithm to transform the image into a texture spectrum is described below: $s\left(m,n\right)\to h\left(k\right)$: (1) the binary image $s\left(m,n\right)$ is interpreted as a binary array $\left\{s_{mn}\right\}$, where the white pixels are 1 s and the black pixels are 0 s; (2) the observation window size W=I×J is selected; (3) by moving the observation window pixel by pixel over the entire matrix $\left\{s_{mn}\right\}$, a binary pattern P is detected for each position on the image under study; (4) the texture unit k is calculated for each pattern P and then the unit k is used as an index in the discrete histogram $h\left(k\right)$ (k=0,1,2,…,K−1), whose length is between 0 and K−1; and (5) the histogram $h\left(k\right)$ is divided by the total texture units $P_{p}$, obtaining the function of probability densities:

(14) $$p_{I\times J}\left(k\right)=\frac{h(k)}{P_{p}}=\frac{h(k)}{\left(M-I+1)(N-J+1\right)}$$

The function $p_{I\times J}\left(k\right)$ is called Texture Spectrum based on the Parallel Encoded Texture Unit (TS_PETU) because the unit k is encoded in parallel, the index I×J indicates the observation window size, and the equalized histogram TS_PETU shows the frequency of occurrence of the texture units calculated from the digital image $\left\{s_{mn}\right\}$. The Algorithm 1: TS_PETU to calculate the TS_PETU histogram is shown below.
**Algorithm 1:** TS_PETUBeginning  Input:  s(m,n) ← user Binary image to transform  I and J ← user Observation window size selection  Calculus:   M and N ←s(m,n) Binary image size    for m:M Displacement over image lines    for m:NDisplacement over image columns  Texture unit calculation:      P←s(m,n) Extraction of binary pattern P from image s(m,n)     $c_{1},c_{2},\ldots ,c_{I}\leftarrow P$ Calculation of decimal values by conversion BCD      $k=c_{1}+c_{2}+,\ldots ,{+c}_{I}$  Texture unit calculation k  Texture unit mapping k to the discrete histogram $h_{I\times J}(k)$      $h_{I\times J}(k)\leftarrow k$ Unit k is assigned to the histogram $h_{I\times J}(k)$      end    end  Calculation of probability density function $p_{I\times J}(k)$ or histogram TS_PETU     $p_{I\times J}(k)\leftarrow h_{I\times J}(k)$ Histogram calculation TS_PETU, $p_{I\times J}(k)$    End

**Figure 4:**
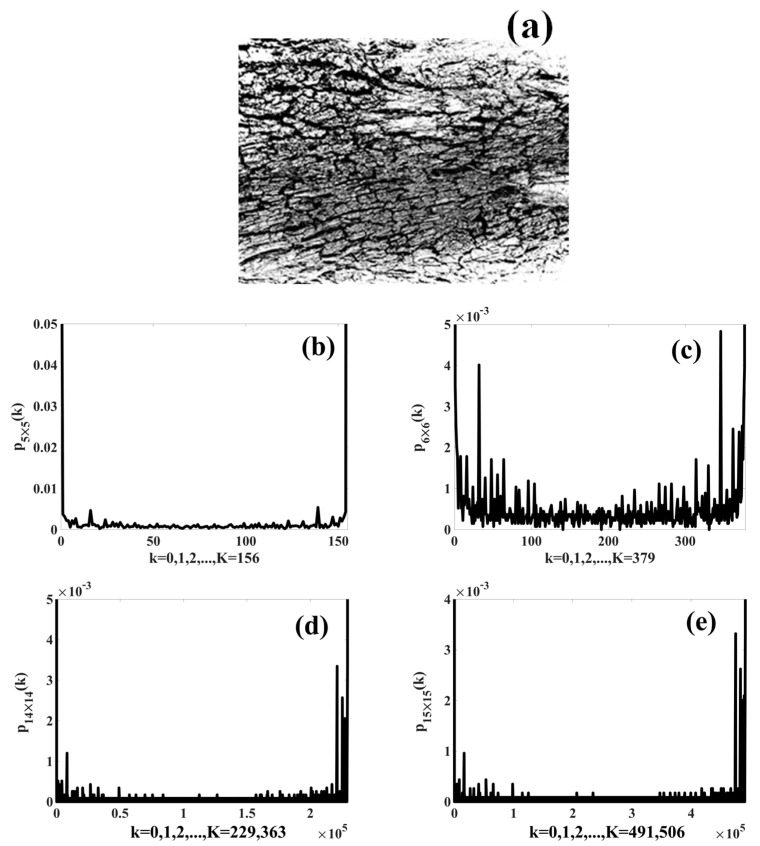
(**a**) Binary image of tree stem used to calculate its texture spectra $p_{I\times J}(k)$; (**b**) $p_{5\times 5}(k)$ calculated with W=5×5; (**c**) $p_{6\times 6}(k)$ calculated with W=6×6; (**d**) $p_{14\times 14}(k)$ calculated with W=14×14; (**e**) $p_{15\times 15}(k)$ calculated with W=15×15.

By applying the described algorithm and the Algorithm 1: TS_PETU, and using different observation window sizes, the histogram TS_PETU was calculated for a binary digital image; see Figure 4. 

Figure 4a shows the binary image $s\left(m,n\right)$ of a tree stem, and its size is M×N=4169×3120 pixels. Figure 4b,c show the texture spectra calculated with observation windows of 5×5 and 6×6 pixels, respectively. Both TS_PETU histograms have low-dimensional space since $R^{156}$ and $R^{379}$ are the respective numbers of dimensions and Conditions (10) and (11) are met. On the other hand, Figure 4d,e show the spectra calculated with windows with 14×14 and 15×15 pixels, respectively. Both TS_PETU histograms have high-dimensional space because the number of dimensions is $R^{229,363}$ and $R^{491,506}$, respectively, and as a consequence, Conditions (12) and (13) are satisfied. 

Based on the results shown in Figure 4, the binary image s(m,n) was transformed into the TS_PETU texture spectrum, which can operate with low- and high-dimensional space. Its region of operation depends on the selected observation window size. 

### 2.5. Texture Information (Entropy)

Knowing that the transformed TS_PETU generates the odds function $p_{I\times J}(k)$, and knowing that in the information theory the amount of information is measured based on a function of probability densities, then by applying the information theory, the amount of texture information extracted from the image can be measured.

Since the texture unit k is a discrete random variable, let us assume that its initial indeterminacy is equal to $k_{\alpha }$ $\left(\alpha =0,1,2,\ldots ,K-1\right)$, where there are K possible states due to dimensional space $R^{K}$, and if we consider that all states are equiprobable, the information provided by the texture unit is [21]

(15) $$I\left(k_{\alpha }\right)={log}_{2}\left(\frac{1}{k_{\alpha }}\right)=-{log}_{2}\left(k_{\alpha }\right)$$

such that the information provided by all the texture units is estimated with

(16) $$I\left(k_{0},k_{1},k_{2},\ldots ,k_{K-1}\right)=I\left(k_{0}\right)+I\left(k_{1}\right)+I\left(k_{2}\right)+\cdots +I\left(k_{K-1}\right)$$

Combining Equations (15) and (16), the texture information is expressed by

(17) $$I\left(k_{0},k_{1},k_{2},\ldots ,k_{K-1}\right)=-{log}_{2}\left(k_{0}\right)-{log}_{2}\left(k_{1}\right)-{log}_{2}\left(k_{2}\right)-\cdots -{log}_{2}\left(k_{K-1}\right)$$

Since the texture unit has probability $p_{k}(k)$, the amount of texture information is obtained by means of the weighted sum

(18) $$H=-p_{0}\left(k\right){log}_{2}\left(p_{0}\left(k\right)\right)-p_{1}\left(k\right){log}_{2}\left(p_{1}\left(k\right)\right)-p_{2}\left(k\right){log}_{2}\left(p_{-}\left(k\right)\right)-\cdots -p_{K-1}\left(k\right){log}_{2}\left(p_{K-1}\left(k\right)\right)$$

Finally, the amount of average texture information is the weighted average value of the amount of texture information of the various states of the unit k, and this is determined with Probability Function (14).

(19) $$H=-\sum_{\alpha =0}^{K-1}p_{I\times J}\left(k\right){log}_{2}\left(p_{I\times J}\left(k\right)\right)=\sum_{\alpha =0}^{K-1}p_{I\times J}\left(k\right){log}_{2}\left(\frac{1}{p_{I\times J}\left(k\right)}\right),$$

Note that, in the limit of the summation, we have the parameter K. Considering the dimensional space of the TS_PETU transform (Equation (8)) in Expression (19), the amount of texture information is calculated through

(20) $$H=-\sum_{\alpha =0}^{I\left(2^{J}-1\right)}p_{I\times J}\left(k\right){log}_{2}\left(p_{I\times J}\left(k\right)\right)$$

**Figure 5:**
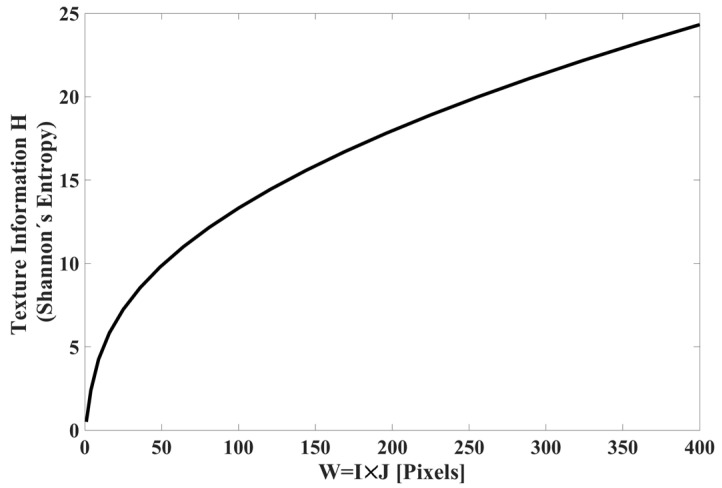
Amount of texture information behavior (Shannon’s entropy) H vs. window size I×J, with an interval of I×J=3×3 to I×J=20×20.

That is, the amount of texture information H is a function of the observation window size w=I×J. To illustrate what has been mentioned, let us consider that all texture units are equiprobable, and as a consequence, Expression (20) can be rewritten as follows:

(21) $$H=-\sum_{\alpha =0}^{I\left(2^{J}-1\right)}\frac{1}{I\left(2^{J}-1\right)+1}{log}_{2}\left(\frac{1}{I\left(2^{J}-1\right)+1}\right),$$

whose behavior is shown in Figure 5.

Observing Figure 5, the y-axis corresponds to the amount of texture information, which can also be identified as the Shannon entropy; the x-axis corresponds to the size of the observation window; and the behavior of H vs. I×J has exponential growth. We can conclude that the amount of texture information extracted from image S is a function of the window size; the larger the window, the more information is extracted. That is, the TS_PETU transform is more efficient in image classification when the window W=I×J it is bigger.

### 2.6. Application

**Figure 6:**
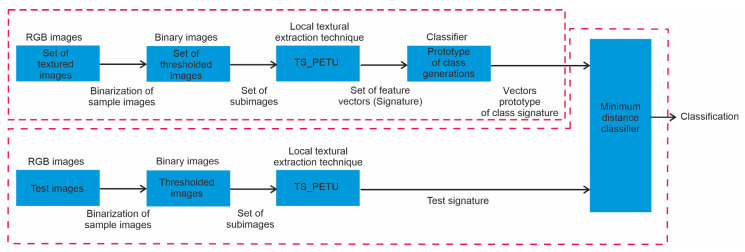
Multi-class classifier based on the image statistic.

Various classifiers have been reported in the literature to which texture spectra are applied as a multidimensional characteristic vector [13,14,15,16,17,18,19,20]. In particular, in references [12,14], a multi-class classifier based on image statistics is described, applied, and optimized. Due to its experimentally demonstrated efficiency, in this section, the TS-PETU texture spectrum is applied as the characteristic vector. In Figure 6, the classifier for multiple classes is schematically shown.

As shown in Figure 6, the classifier consists of two stages: learning and recognition. In the learning stage, the digital images are classified by a human expert, each image is considered a class, and in the database there are C classes. To characterize each class based on its local textural characteristics, for each class $S_{c}\left(m,n\right)$ $\left(c=1,2,\ldots ,C\right),$ a series of subimages is drawn at random $S_{c,s}\left(m,n\right)$ $\left(s=1,2,\ldots ,S\right)$ and then for each subimage $S_{c,s}\left(m,n\right)$, the texture spectrum is calculated $p_{I\times J}^{c,s}\left(k\right)$. Finally, the characteristic vector of class c is denoted by $p_{I\times J}^{c}\left(k\right)$ and is determined with the average of the texture spectra $p_{I\times J}^{c,s}\left(k\right)$, $p_{I\times J}^{c}\left(k\right)=\frac{1}{S}\sum_{s=1}^{S}p_{I\times J}^{c,s}\left(k\right)$ [12,14]. In the recognition stage, from a test image $S_{t}\left(m,n\right)$, a series of subimages is randomly extracted $S_{t,p}\left(m,n\right)$ $\left(p=1,2,\ldots ,P\right)$. For each subimage $S_{t,p}\left(m,n\right)$, the texture spectrum is calculated $p_{I\times J}^{t,p}\left(k\right)$, and then the characteristic vector of the test image $p_{I\times J}^{t}\left(k\right)$ is obtained with the average of the texture spectrum $p_{I\times J}^{t,p}\left(k\right)$, $p_{I\times J}^{t}\left(k\right)=\frac{1}{P}\sum_{p=1}^{P}p_{I\times J}^{t,p}\left(k\right)$. Finally, the test image $S_{t}\left(m,n\right)$ is classified using the minimum distance between the prototype vector $p_{I\times J}^{c}\left(k\right)$ and the test image vector $p_{I\times J}^{t}\left(k\right)$ [12].

## 3. Experimental Work

In this experimental work, two digital image databases were used and three series of experiments were developed. One database was provided by Interceramic^®®^, and the second database was acquired in natural environments. In the first experiment, each image from the Interceramic^®®^ database was binarized using a global threshold technique. Subsequently, each binary image had its local texture characteristics extracted by means of the TS_PETU transform, and then the texture information was measured. With the results, the H vs. I×J behavior graph was created. In the second experiment, the digital images from the Interceramic^®®^ database were classified, the TS_PETU histogram was used as a characteristic vector in the classifier described in Section 2.6, and with the results, the following behavior graphs were constructed: classification efficiency $({Ef}_{I\times J})\;vs.$ window size (W=I×J), sorting efficiency $({Ef}_{I\times J})\;vs.$ dimensional space $\left(R^{K}\right)$, and sorting efficiency $({Ef}_{I\times J})\;vs.$ texture information (H). In the third series of experiments, the TS_PETU histogram was used again as a feature vector in the classifier described in Section 2.6, and then a database of natural images of tree stems was classified. With the results of this series of experiments, the following behavior graphs were again generated: classification efficiency $({Ef}_{I\times J})\;vs.$ window size (W=I×J), sorting efficiency $({Ef}_{I\times J})\;vs.$ dimensional space $\left(R^{K}\right)$, and sorting efficiency $({Ef}_{I\times J})\;vs.$ texture information (H). Finally, the numerical experiments were implemented by applying the MatLab 2016b software and a GHIA computer with the following characteristics: Intel(R) Core (TM) i7-4790 CPU 3.60 GHz processor and 8GB RAM.

### 3.1. Experiment 1: Measurement of Texture Information Based on TS_PETU

**Figure 7:**
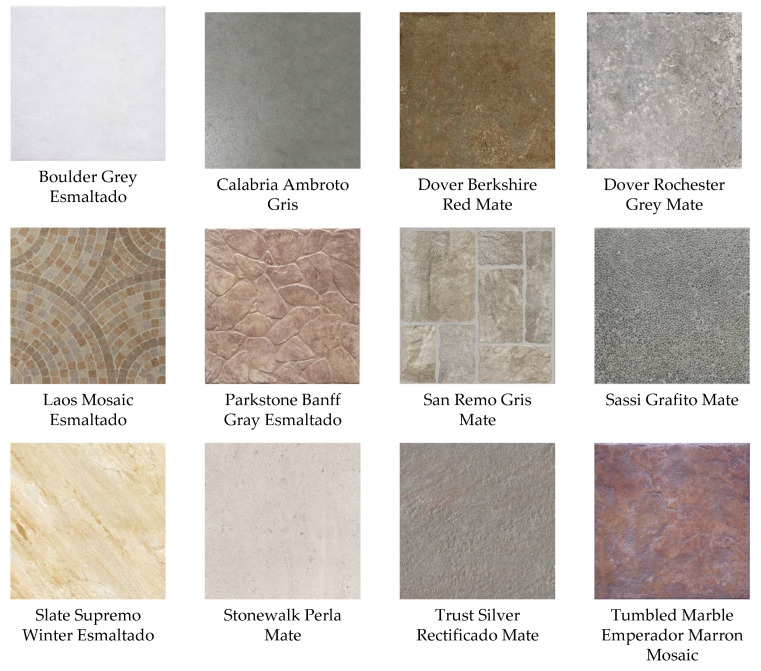
Ceramic tiles produced by Interceramic^®®^.

**Figure 8:**
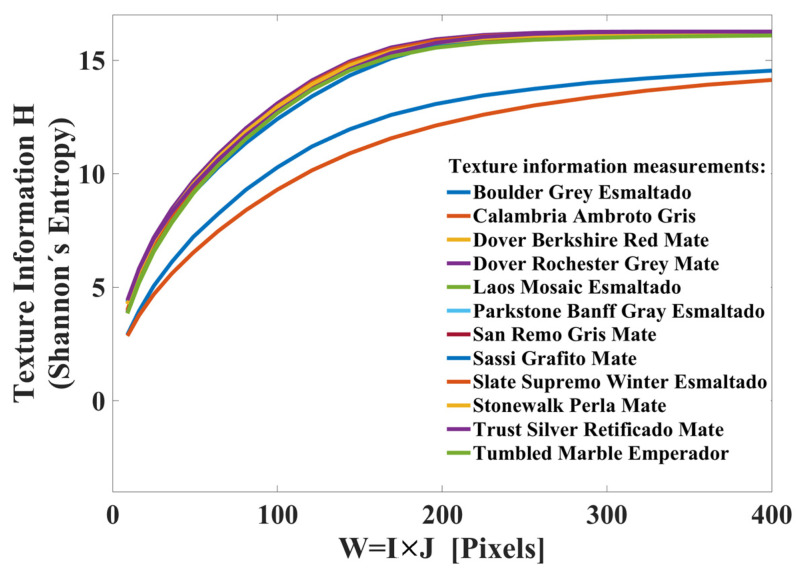
Experimental texture information measured from Interceramic^®®^’s database.

As a first step, the Interceramic^®®^ image database was taken and the digital images were binarized by applying a global threshold technique. The RGB images are observable in Figure 7, and each image has a size of M×N=300×300. Using a window size within the interval of I×J=3×3 to I×J=20×20, local texture features were extracted from the binary image, and then the amount of texture information H in the histogram $p_{I\times J}\left(k\right)$ was calculated. Next, using the measurements of *H* and the window size, the behavior graph of H vs. W=I×J was created (see Figure 8).

As can be observed in Figure 8, the texture information *H* grows when the observation window size W=I×J is bigger. The behavior graph of H vs. I×J has an exponential form, and its behavior is similar for all images. The minimum texture information value correspondents to “Calabria Ambroto Gray” and its value is $H_{3\times 3}=2.86$. The maximum texture information value corresponds to “Dover Rochester Grey Mate” and the value is $H_{20\times 20}=16.26$. Then, by comparing Figure 5 (theoretical results) with Figure 8 (experimental results), it can be seen that the theory and the experiments are in accordance. We can then infer that the image classification efficiency with the TS_PETU transform is better when the observation window is larger. This is corroborated with two series of image classification experiments, whose results are shown in the following sections. 

During the computing process, the execution time and the number of operations were measured in the calculation of the histogram $p_{I\times J}\left(k\right)$. Table 1 was generated with the measurements. By analyzing Table 1, it can be seen that the execution time increases due to the increase in the size of the observation window. The increase in time is attributed to the fact that the number of computational operations also grows with the window size. In our experiments, the minimum execution time measured was 0.3770 s for window I×J=3×3 and the number of computational operations was 1,509,668. On the other hand, the maximum execution time was 0.6390 s, the window size was I×J=20×20 pixels, and the number of operations was 63,089,839.

### 3.2. Experiment 2: Classification of Database Images from Interceramic^®®^

In this section, the digital image database provided by Interceramic^®®^ was classified. The tiles were manufactured in their industrial plant and were analyzed and named by a human expert (see Figure 7). In addition, the ceramic tiles were photographed at Interceramic^®®^ under controlled lighting conditions, rotation, and scale. The size of the images is 300 × 300 pixels. On the other hand, by calculating the TS_PETU histogram with window sizes within the interval of I×J=3×3 to I×J=20×20 and using the TS_PETU histogram as a characteristic vector in the multi-class classifier (see Section 2.6), the Interceramic^®®^ database was identified. In the classifier, the images used in the learning stage were the same images as those used in the recognition stage. In addition, the subimage size was similar for both stages. In Table 2, the parameters for the classifier are shown.

The results obtained in the classifier are expressed through a confusion matrix $M_{c}$, the main diagonal of which is the correct identification [12], the elements outside the main diagonal are classification errors, the rows correspond to the test images, and the columns correspond to the prototype images (master images). Table 3 shows an example of confusion matrix $M_{c}$, which was obtained in our experimental work. In this example, the TS_PETU histogram was calculated using a I×J=5×5 pixel observation window.

From the confusion matrix of the example (Table 3), the classification efficiency for the texture spectrum $p_{5\times 5}(k)$ is calculated by

(22) $${Ef}_{I\times J}=\frac{\sum diag(M_{c})}{C}\times 100,$$

where ${Ef}_{I\times J}$ is the classification efficiency in terms of percentage, the term I×J is the observation window size, $\sum diag(M_{c})$ is the sum of all of the elements of the main diagonal (marked in blue) in the matrix $M_{c}$, and C is the total number of classes. Considering the values of the confusion matrix (Table 3) and the parameters of Table 2, the efficiency ${Ef}_{5\times 5}$ is

(23) $${Ef}_{5\times 5}=\frac{1+1+1+1+1+1+1+1+1+1+1+1}{12}\times 100=100\%$$

**Figure 9:**
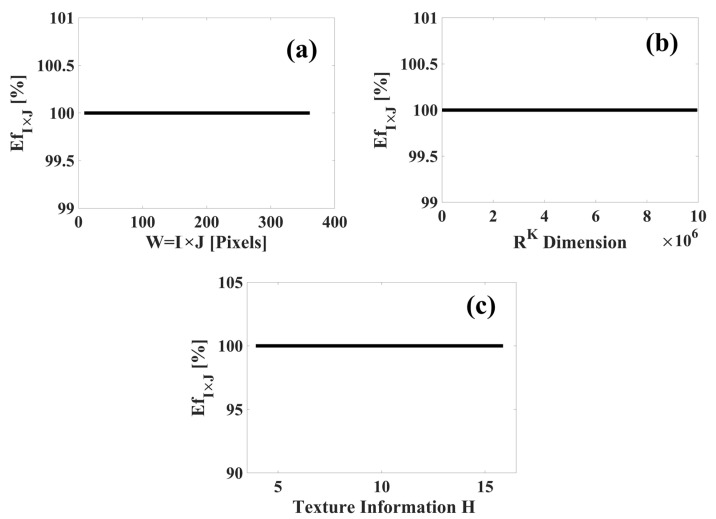
Experimental results obtained in the classification of images of ceramic tiles: (**a**) classification efficiency $({Ef}_{I\times J})\;vs.$ window size (W=I×J); (**b**) classification efficiency $({Ef}_{I\times J})\;vs.$ dimensional space $\left(R^{K}\right)$; (**c**) classification efficiency $({Ef}_{I\times J})\;vs.$ texture information (H).

Based on Equation (23), the classification efficiency of the texture spectrum $p_{5\times 5}(k)$ is equal to ${Ef}_{5\times 5}=100\%$. The experimental results are shown in Figure 9.

In Figure 9, the classification efficiency for the ceramic tile images is 100%. This high efficiency can be attributed to the following points:

1. The images of the ceramic tiles were acquired under controlled conditions, such as: lighting, scale, rotation, and translation. This increased the possibility of success in the identification of images, and as a consequence, also reduced possible classification errors.
2. The TS_PETU texture extraction technique correctly characterized the digital image through its local texture characteristics—that is, the TS_PETU transform extracted sufficient texture information to achieve high image classification efficiency.
3. The statistical classifier for multiple classes was optimized to achieve high image identification efficiency. Optimization was achieved based on the size of the subimages and the number of subimages S and *P*.

The TS_PETU transform must be calculated with a window size within the interval of I×J=3×3 to I×J=19×19, since if the window has a size of I×J=20×20 or bigger, there is an overflow of physical memories. However, with these results, it is confirmed that the TS_PETU transformed has potential application in digital images and surface quality control and can be operated in low- or high-dimensional space.

### 3.3. Experiment 3: Classification of Natural Images

**Figure 10:**
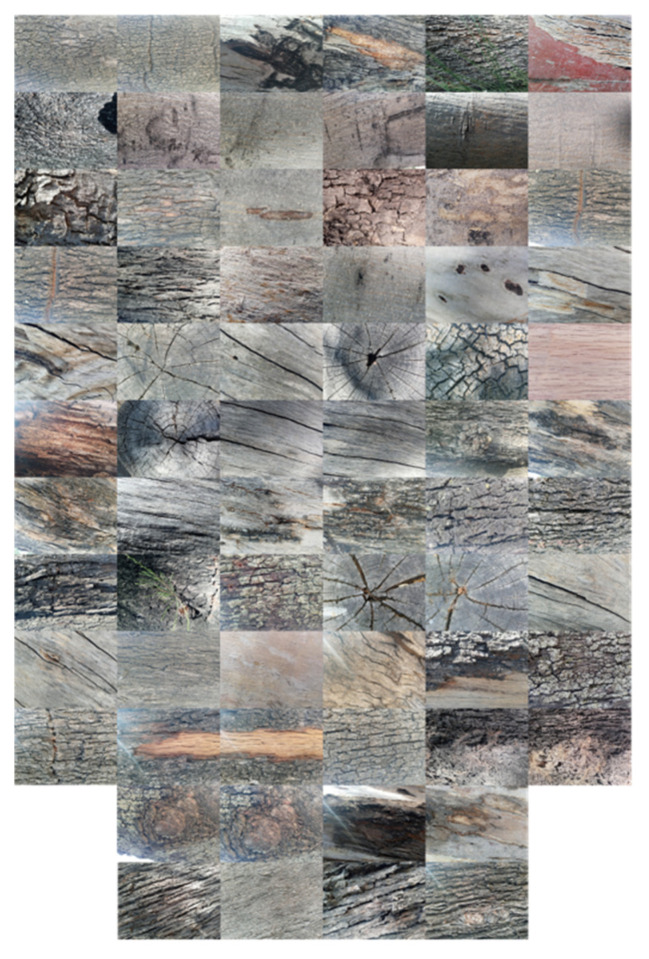
Natural image database used in the numerical experiments.

In this section, using the TS_PETU histogram as a characteristic vector in the classifier described in Section 2.6 and with window sizes of I×J=3×3 to I×J=10×10, a database of 64 natural images was classified. The images were of trees, they were RGB, they were acquired using an LG-Q50 camera, and the rotation, scale, and translation were controlled but the lighting was natural. The size of each image is 3120 × 4260 pixels and the images can be observed in Figure 10.

On the other hand, the characteristics of the classifier were based on the data in Table 4, where the number of classes is C=68, the number of subimages is S=100, and the subimage size is 1560×2130.

**Figure 11:**
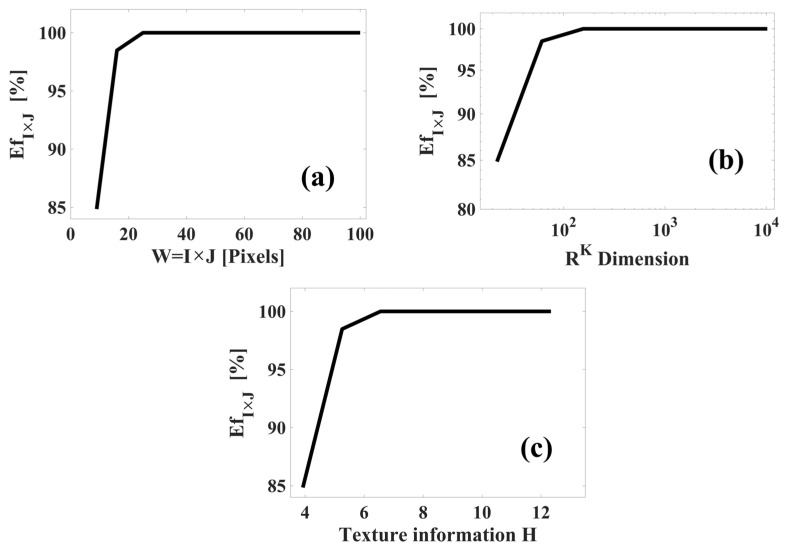
Experimental results obtained in the classification of natural images of trees: (**a**) ${Ef}_{I\times J}$ vs. W=I×J; (**b**) ${Ef}_{I\times J}$ vs. $R^{K}$ dimension; (**c**) ${Ef}_{I\times J}$ vs. texture information (H).

Again, the results obtained in the classification of natural images are expressed through the confusion matrix $M_{c}$. As previously mentioned, the main diagonal is the correct identification, the elements outside of the main diagonal are classification errors, the rows correspond to the test images, and the columns correspond to the master images. Finally, the classification efficiency ${Ef}_{I\times J}$ was calculated with Equation (22). The experimental results are observable in Figure 11, where the behavior graphs of ${Ef}_{I\times J}$ vs. W=I×J, ${Ef}_{I\times J}$ vs. $R^{K}$, and ${Ef}_{I\times J}$ vs. H are shown.

As can be observed in Figure 11, the efficiency ${Ef}_{I\times J}$ increases with window size growth W, dimensional space $R^{K}$, and texture information H. According to these results, the spectrum TS_PETU has an efficiency of ${Ef}_{I\times J}=100\%$ when W=I×J=5×5 or bigger, there is a dimension of $R^{156}$ or more, and the texture information is H=6.25 or more. This high efficiency can be attributed to points 2 and 3, which were mentioned in Section 3.2. On the other hand, the lowest efficiency was measured in ${Ef}_{I\times J}=84.84\%$, where W=I×J=3×3, there is a dimension of $R^{22},$ and the texture information is H=3.92. The classification error is attributed to the lighting conditions not being controlled during the image acquisition process. However, from these results, the following can be concluded: The TS_PETU transform has high efficiency in image recognition if it is carried out in a region of low-dimensional space, (see Equations (10)–(13) and Figure 3). In addition, our proposal has potential application in the recognition of images in natural environments since its efficiency is very high when the window W is of a larger size.

## 4. Discussion

In this work, a new texture extraction technique is proposed, which is called Texture Spectrum based on the Parallel Encoded Texture Unit (TS_PETU) because the texture unit is calculated based on a parallel coding. The TS_PETU technique transforms a binary image into a probability density function (equalized histogram) in terms of texture units. The equalized histogram can be calculated using windows greater than 3×3 and the histogram is in dimensional space. Its efficiency in the extraction of texture information and image classification was verified by conducting three series of experiments: In the first series, it is corroborated that the amount of texture information depends on the window size W; in the second series, its efficiency is confirmed when the images are acquired under controlled conditions; and in the third series, its efficiency is verified when the images are acquired under non-controlled lighting conditions. Based on the results obtained, the following points can be inferred:

1. Texture Spectrum based on the Parallel Encoded Texture Unit (TS_PETU) represents a binary image $s\left(m,n\right)$ as a probability density function in terms of texture units $p_{I\times J}\left(k\right)$, whose characteristics are low-dimensional space and high efficiency of image classification.
2. The TS_PETU histogram shows the frequency of occurrence of the texture units calculated from the binary image under study.
3. Because the texture unit is calculated by applying parallel coding concepts, the TS_PETU histogram has low-dimensional space and it is possible to use large windows.
4. The amount of texture information contained in the TS_PETU histogram is based on the observation window size W=I×J.
5. It is experimentally corroborated that the TS_PETU transform has high efficiency in image classification.
6. Classification efficiency is improved using windows W with larger sizes; see Section 3.1, Section 3.2 and Section 3.3.
7. The TS_PETU histogram can work in low- and high-dimensional space regions, and in both regions there can be high image classification efficiency.
8. The efficiency of the TS_PETU transform increases when the conditions are controlled during the image acquisition process.
9. The TS_PETU histogram can be calculated using parallel compute.
10. The classification efficiency with the TS_PETU transform can be reduced due to noise produced by the illumination source and the electronic systems used during image acquisition and processing due to numerical computational errors and retroflections generated on the surface of the material under study [22].
11. The TS_PETU transform has significant practical application, and some benefits for the user are high efficiency, short execution times, low-dimensional space, selectivity through the observation window, implementation with parallel computing, and easy implementation with electronic cards.

Comparing TS_PETU with the techniques reported in reference [11], our proposal can be estimated with observation windows greater than 3×3 and its low-dimensional space can be conserved, the amount of texture information increases with the window size, and as a consequence, the classification efficiency is improved. Now, comparing the TS_PETU with the original versions of the LBP and CCR transforms, which are based on BCD coding, our proposal offers some advantages, as can be seen in Table 5.

When the images are acquired under controlled conditions, the three transforms have high efficiency greater than 90%. However, in our experiments, when the images are acquired in natural environments, the CCR transform has an efficiency of less than 80%, the LBP has an efficiency of 98.40, and the TS_PETU has an efficiency of up to 100 if the observation window is equal to or greater than I×J=5×5. Two important points to highlight are that (1) the CCR and LBP cannot work with windows larger than I×J=5×5 because the dimensional space is very large and an overflow is generated in the computer, whereas the TS_PETU can operate with windows within the interval of I×J=3×3 to I×J=19×19 and the computational overflow is generated for the window I×J=20×20, and (2) the decrease in efficiency is attributed to the fact that the conditions were not controlled during the acquisition of the images.

Due to the definition of the texture unit and the efficiency obtained experimentally, our future research lines are (1) to apply parallel compute to reduce execution times, (2) to develop artificial vision applications, (3) to describe the mathematical foundation of the TS_PETU transform, and (4) to optimize image classification with the TS_PETU transform and perform a sensitivity analysis.

## 5. Conclusions

In this work, a new texture extraction technique is proposed and applied that is called Texture Spectrum based on the Parallel Encoded Texture Unit (TS_PETU) because the unit k is calculated based on parallel coding. The TS_PETU transformation is based on a local analysis of the binary image s(m,n), and this represents the image as a probability density function $p_{I\times J}(k)$ in terms of texture units. The main characteristics of the function $p_{I\times J}(k)$ are: (1) It can be calculated with windows greater than W=I×J=3×3, (2) it has very low dimensional space $R^{I\left(2^{J}-1\right)+1}$, (3) the amount of texture information H is based on the window size I×J, and (4) it has high efficiency in image classification since it can reach up to 100%. Experimentally, the four characteristics were verified, confirming that the TS_PETU transform has high efficiency in image recognition.

The TS_PETU transform can be implemented in real time due to the parallel coding of the texture unit. In addition, it has potential industrial application when the surface or texture characteristics are important.

## Figures and Tables

**Table 1:** Average execution time and number of operations required to calculate the histogram $p_{I\times J}\left(k\right)$ for tile images from Interceramic^®®^, M×N=300×300 pixels.

Window Size I×J Pixels	Number of Operations	Runtime, Seconds
3×3	1,509,668	0.3770
4×4	2,734,479	0.3770
5×5	4,293,184	0.3770
6×6	6,178,775	0.3770
7×7	8,384,292	0.3770
8×8	10,902,823	0.3780
9×9	13,727,504	0.3800
10×10	16,851,519	0.3810
11×11	20,268,100	0.3860
12×12	23,970,527	0.3880
13×13	27,952,128	0.3890
14×14	32,206,279	0.3900
15×15	36,726,404	0.3980
16×16	41,505,975	0.4060
17×17	46,538,512	0.4310
18×18	51,817,583	0.4370
19×19	57,336,804	0.4670
20×20	63,089,839	0.6390

**Table 2:** Parameters used in the classifier for multiple classes.

Learning Stage		Recognition Stage	
Number of classes, C	C=12	Test images number, T	T=12
Image size, $S_{c}\left(m,n\right)$	M×N=300×300	Test image size, $S_{t}\left(m,n\right)$	M×N=300×300
Number of subimages, $S_{c,s}\left(m,n\right)$	S=100	Number of subimages, $S_{t,p}\left(m,n\right)$	P=100
Subimage size, $S_{c,s}\left(m,n\right)$	150×150	Subimage size, $S_{t,p}\left(m,n\right)$	150×150

**Table 3:** Confusion matrix $M_{c}$ obtained using a 5 × 5 window size.

	Master Images												
		Boulder Grey	Calabria Ambroto	Dover Bershire	Dover Rochester	Laos Mosaic	Parkstone Banff	Sam Remo	Sassi Grafito	Slate Supremo	Stonewalk Perla	Trust Silver	Tumber Marble
**Test Images**	**Boulder Grey**	1	0	0	0	0	0	0	0	0	0	0	0
	**Calabria Ambroto**	0	1	0	0	0	0	0	0	0	0	0	0
	**Dover Bershire**	0	0	1	0	0	0	0	0	0	0	0	0
	**Dover Rochester**	0	0	0	1	0	0	0	0	0	0	0	0
	**Laos Mosaic**	0	0	0	0	1	0	0	0	0	0	0	0
	**Parkstone Banff**	0	0	0	0	0	1	0	0	0	0	0	0
	**Sam Remo**	0	0	0	0	0	0	1	0	0	0	0	0
	**Sassi Grafito**	0	0	0	0	0	0	0	1	0	0	0	0
	**Slate Supremo**	0	0	0	0	0	0	0	0	1	0	0	0
	**Stonewalk Perla**	0	0	0	0	0	0	0	0	0	1	0	0
	**Trust Silver**	0	0	0	0	0	0	0	0	0	0	1	0
	**Tumber Marble**	0	0	0	0	0	0	0	0	0	0	0	1
	Classification efficiency												100%

**Table 4:** Parameters used in the classifier for multiple classes.

Learning Stage		Recognition Stage	
Number of classes, C	C=68	Number of test images, T	T=68
Image size, $S_{c}\left(m,n\right)$	M×N=3120×4260	Test image size, $S_{t}\left(m,n\right)$	M×N=3120×4260
Number of subimages, S	S=100	Number of subimages, $S_{t,p}\left(m,n\right)$	S=100
Subimage size, $S_{c,s}\left(m,n\right)$	1560×2130	Subimage size, $S_{t,p}\left(m,n\right)$	1560×2130

**Table 5:** Comparative table between TS_PETU and the original versions of the CCR and LBP.

Interceramic®® Image Database					
TS_PETU		CCR		LBP	
Window Size	Efficiency (%)	Window Size	Efficiency (%)	Window Size	Efficiency (%)
3×3	100	3×3	94.23	3×3	100
4×4	100	4×4	99.23	4×4	Not applicable [23,24]
5×5	100	5×5	Overflow	5×5	Overflow
6×6	100	6×6		6×6	
7×7	100	7×7		7×7	
8×8	100	8×8		8×8	
9×9	100	9×9		9×9	
10×10	100	10×10		10×10	
11×11	100	11×11		11×11	
12×12	100	12×12		12×12	
13×13	100	13×13		13×13	
14×14	100	14×14		14×14	
15×15	100	15×15		15×15	
16×16	100	16×16		16×16	
17×17	100	17×17		17×17	
18×18	100	18×18		18×18	
19×19	100	19×19		19×19	
20×20	Overflow	20×20		20×20	
Natural image database					
TS_PETU		CCR		LBP	
Window size	Efficiency (%)	Window size	Efficiency (%)	Window size	Efficiency (%)
3×3	84.848	3×3	78.083	3×3	98.40
4×4	98.484	4×4	87.030	4×4	Not applicable [23,24]
5×5	100	5×5	Overflow	5×5	Overflow
6×6	100	6×6		6×6	
7×7	100	7×7		7×7	
8×8	100	8×8		8×8	
9×9	100	9×9		9×9	
10×10	100	10×10		10×10	

## Data Availability

The data related to the results that support our conclusions are available upon request to the authors. It can be done via e-mail. We will be pleased to respond.
